# Disparities in high risk prenatal care adherence along racial and ethnic lines

**DOI:** 10.3389/fgwh.2023.1151362

**Published:** 2023-07-25

**Authors:** Molly M. Stegman, Elizabeth Lucarelli-Baldwin, Serdar H. Ural

**Affiliations:** ^1^ College of Medicine, The Pennsylvania State University, Hershey, PA, United States; ^2^ Department of Obstetrics and Gynecology, College of Medicine, The Pennsylvania State University, Hershey, PA, United States

**Keywords:** obstetrics, ethnicity, race, no-show, minority, maternal fetal medicine, prenatal care

## Abstract

The term “high-risk pregnancy” describes a pregnancy at increased risk for complications due to various maternal or fetal medical, surgical, and/or anatomic issues. In order to best protect the pregnant patient and the fetus, frequent prenatal visits and monitoring are often recommended. Unfortunately, some patients are unable to attend these appointments for various reasons. Moreover, it has been documented that patients from ethnically and racially diverse backgrounds are more likely to miss medical appointments than are Caucasian patients. For instance, a case-control study retrospectively identified the race/ethnicity of patients who no-showed for mammography visits in 2018. Women who no-showed were more likely to be African American than patients who kept their appointments, with an odds ratio of 2.64 (4). Several other studies from several other primary care and specialty disciplines have shown similar results. However, the current research on high-risk obstetric no-shows has focused primarily on *why* patients miss their appointments rather than *which patients* are missing appointments. This is an area of opportunity for further research. Given disparities in health outcomes among underrepresented racial/ethnic groups and the importance of prenatal care, especially in high-risk populations, targeted attempts to increase patient participation in prenatal care may improve maternal and infant morbidity/mortality in these populations.

## Introduction

Approximately 6%–8% of all pregnancies in the United States are considered high-risk pregnancies (1). The term “high-risk pregnancy” describes a pregnancy in which the pregnant patient and the fetus are at an increased risk for complications. Risk factors include chronic health conditions such as diabetes or hypertension, obesity, multiple births, and young or old maternal age (2). While some of these conditions can be resolved prior to pregnancy, others cannot and must be monitored and treated during pregnancy. In order to best protect the pregnant patient and the fetus, the patient should be seen frequently for prenatal care and monitoring.

Unfortunately, for various reasons, regularly attending these prenatal appointments can be challenging for many patients, and the no-show rate reflects this. While there has been research into the reasons for no-shows in high-risk obstetric clinics, there has not been research to identify which patient populations are at an increased risk for no-shows. Knowing that racial and ethnic minorities have higher rates of no-shows in other specialties, it seems likely that racial and ethnic minorities also have higher rates of no-shows in high-risk obstetric clinics. Because there is no data on racial and ethnic disparities of no-show rates in general obstetrics or in high-risk obstetric clinics, this review will explore the existing literature relevant to racial and ethnic disparities of no-show rates in various other specialties. Additionally, this review will explore the existing literature on no-show rates in high-risk obstetric clinics.

## Methods

A literature search utilizing PubMed was conducted to identify sources relevant to either racial and/or ethnic disparities of no-show rates or high-risk obstetrical no-show rates. There was no restriction on publication date. The search terms used were race, ethnicity, disparities, differences, high-risk obstetrics, high-risk pregnancy, no-show, and missed appointments. Studies that were not relevant to either topic or were written in a language other than English were excluded. The reference sections of the included sources were used to identify other relevant literature.

## Race and ethnicity in no-shows

It has been documented that patients from ethnically and racially diverse backgrounds are more likely to miss medical appointments. A cross-sectional study done in 2016 sought to determine the relationship between race and ethnicity and missed appointments. It was known that missed appointments are associated with factors such as lower socioeconomic status and medical complexity, but an association between race/ethnicity had not been established. As such, this study aimed to fill that research gap. The chart review found that the overall no-show rate between 44 specialty and primary care clinics was 23%, ranging from 0% to 58%. This rate was significantly higher for all patients who were non-White. Rates of missed appointments were 1.8 times higher for African Americans, 2 times higher for American Indian/Alaskan Natives, and 2 times higher for Latino/Hispanic patients when compared to non-White non-Hispanic patients (3).

Several other studies have found a similar association between race/ethnicity and increased no-show rates. These studies were specialty-specific and are broken down by specialty in this review.

### Mammography

A case-control study retrospectively identified the race/ethnicity of patients who no-showed for mammography visits in 2018. Between January and March of 2018, 5,060 patients were scheduled for screening mammograms, and 6.2% were no-shows. Race/ethnicity was recorded in the electronic medical record as non-Hispanic white, Hispanic, Black, Asian, or unknown. Data was reported as non-White Hispanic, African American, or Other. Women who no-showed were more likely to be African American than patients who kept their appointments, with an odds ratio of 2.64. There was no discussion on the likelihood of missing appointments for other races/ethnicities (4).

### Primary care

A retrospective study aimed to determine which patients were no-showing to their primary care appointments and why. This study was done in a primary care clinic that primarily served Latino, immigrant, and low-income patients. The no-show rate was above average at this clinic. Over a five-month period, 7,508 patients were scheduled, and 5,604 were included in the analysis. 16% of these were no-shows. Patient demographics were categorized as Black, Hispanic, White, Asian, and Other. Patients who no-showed were more likely to be Black or Hispanic when compared to White patients. There was no discussion on no-show rates for Asian or Other (5).

### Neurology

A retrospective study aimed to identify predictors of no-shows to neurology clinics. Between July 2013 and September 2018, 71,178 patients were scheduled, and 16% were no-shows. Race was categorized as American Indian/Alaskan Native, Asian, Black/African American, Native Hawaiin/Other Pacific Islander, Other, Unknown, and White. Black/African American patients were more likely to no-show than White/Caucasian patients, with an odds ratio of 1.4712. Asian and American Indian/Alaskan Native patients were less likely to no-show compared to White/Caucasian patients, with an odds ratio of 0.6871. There was no difference in no-show rate odds for Native Hawaiin/Other Pacific Islander (6).

### Pediatrics

Several retrospective studies examined the association between race/ethnicity and pediatric no-show rates. One study quantified the no-show rate across 14 subspecialty pediatric clinics and identified patient characteristics associated with missed appointments. Between January 2013 and December 2018, there were 128,117 appointments, and 18.1% were no-shows. Black race/ethnicity was strongly associated with missed appointments (7).

A study published in 2015 aimed to describe the frequency of missed appointments in pediatric patients with type 1 diabetes and evaluate the relationship between disease control and missed appointments. Over 43 months, 1,002 patients had two or more appointments scheduled. Of these patients, 68% missed no appointments, 17% missed one appointment, and 15% missed two appointments. Patients who were a member of a racial/ethnic minority group were more likely to miss their appointment (8).

Another study aimed to quantify no-show disparities in outpatient surgical care. Among the 10,162 patients between 2017 and 2019, 16% had at least one no-show. Race was categorized as White, Black, Hispanic, or Other. Black race was associated with having at least one no-show, with an adjusted odds ratio of 3.3. (9)

A study published in 2021 examined socioeconomic and imaging exam factors associated with missed pediatric radiology visits. Over a 12-month period, data from the EMR was retrospectively analyzed for 7,275 patients. Patients of the African American race were more likely to miss appointments, with an odds ratio of 1.9 (10).

A survey study published in 2015 aimed to determine factors associated with missing pediatric primary care appointments. 1,537 patients were called, and 386 patients completed the survey. Patients with high no-show rates were placed in one group and compared against the rest. The no-show group had more African American patients (11).

### Orthopedics

A retrospective study examined the association between no-show rates and patient appointment time, and provider characteristics in an orthopedic clinic. In the calendar year of 2016, there were 25,381 appointments scheduled, and 11.5% of these were no-shows. Patients were asked to identify as either White, Black, multiple, or other. Black patients had higher no-show rates than White patients, with an odds ratio of 1.96. Patients who reported other or multiple did not have higher no-show rates (12).

### Ophthalmology

A retrospective case-control study aimed to identify demographic, medical, and socioeconomic characteristics that increase the odds of no-shows for patients with chronic eye conditions in an ophthalmology practice. Between January 2013 to December 2018, there were 106,652 visits. 12.4% were no-shows. Race was divided into White, Black, Asian, American Indian, Native Hawaiin, and Other. Ethnicity was Hispanic or non-Hispanic. Black race and Hispanic ethnicity were found to increase the odds of no-show, with odds ratios of 1.75 and 1.6, respectively. White and Asian races were found to decrease the odds of no-show, with odds ratios of 0.55 and 0.82 (13).

### GI endoscopy

A retrospective observational cohort study evaluated predictors of no-shows in a gastrointestinal clinic. During a 17-month study period, 6,157 patients were scheduled for Gastrointestinal procedures. Of those, 29% were no-shows. African-American race had the highest rate of no-shows when compared to other races. This rate was 32% (14).

## High risk-obstetric no-shows

The current research on high-risk obstetric no-shows primarily focuses on why patients miss their appointments rather than which patients are missing appointments. One study from 1994 was conducted to determine the reasons for missed appointments and the impact of knowledge of diagnosis and the perception of this diagnosis on appointment attendance. 506 women at a high-risk obstetric clinic were called and surveyed after missing their appointment. 118 responses were analyzed. Many women who missed appointments did not know why they were being seen and did not perceive the care as useful (15). With a similar goal, a 2008 study surveyed patients at a high-risk pregnancy clinic regarding reasons for missed appointments. This clinic had a 28% no-show rate, and the study was interested in understanding why. Of the 261 patients included in the study, 41% were reached by telephone. Patients reported difficulty with transportation, scheduling problems, oversleeping, forgetting, and lack of care for their child or sick relative as reasons. Campbell et al. suggested that there may be a socioeconomic link to missed appointments but did not explore this idea further (16).

One study was interested in who was missing the appointments rather than why. A survey study published in 1996 aimed to determine if physicians could predict which patients are missing their high-risk obstetric visits. Feierabend et al. stated that creating interventions to decrease no-shows would be most effective if directed toward the patients who are at greater risk of missing their appointments. Physicians completed surveys at each patient's initial visit, predicting if the patient would have a no-show at their next appointment. It was found that this could not be predicted by the physicians (17). There was no further research to determine if there was a better method for identifying patients at increased risk of missing their high-risk obstetric visits.

## Discussion

This review highlights the need for research on high-risk obstetric no-show rates in racially and ethnically diverse patients. Significant research shows that racial and ethnic minorities are at increased risk of missing medical appointments. Several studies show that African American patients are at increased likelihood of missing appointments across various specialties. Some of these studies also show that Hispanic/Latino patients are also at an increased likelihood and Asian and Pacific Islander patients are at a decreased likelihood when compared to non-Hispanic White patients. It is important to note that there were differences in the categorization of race across the various studies. Some included Native Hawaiin, Pacific Islander, and Native American, while others only offered “other” in addition to African American/Black, White, Asian and Hispanic. Despite collecting the data, several studies did not discuss the results of races other than African American and White. One study grouped Asian and Hispanic as one race due to a lack of data. Therefore, while the majority of conclusions from the studies show increased risk only for the African American race, this could be due to a lack of representation of other races in the study analyses.

These studies have covered the demographics of missed appointments in family medicine, pediatrics, orthopedics, ophthalmology, neurology, and gastroenterology clinics, but not obstetrics and gynecology clinics. Obstetrics and gynecology research has only focused on other aspects of missed appointments thus far, including reasons for missed appointments and the impact of knowledge of the diagnosis and perceived threat. Patients were missing their appointments for reasons such as transportation difficulties, scheduling problems, oversleeping, forgetting, and lack of care for their child or sick relative. Many patients who missed appointments did not know why they were being seen and did not perceive their diagnosis as a threat. If there is a race/ethnicity relation in high-risk obstetric missed appointments, like there is with other specialties, targeted attempts to increase patient participation in prenatal care may improve maternal and infant morbidity/mortality in these populations.

Research that attempted to predict which patients would miss appointments was unsuccessful. This research was published in 1990, and there has not been another attempt to identify patients at increased risk for missing high-risk obstetric visits since.

With these research gaps in mind, there is a need to identify whether racial and ethnic minorities are at an increased risk of missing their high-risk obstetric visits. This would expand on the existing research on the relationship between race/ethnicity and no-show rate and potentially increase high-risk obstetric visit attendance. It is important to note that there are multiple social determinants of health, trust issues, and cultural factors that likely play a larger role in missing appointments than do genetic differences between people of different races and ethnicities. Future research could focus on the interplay of these factors for a truer understanding of barriers to care.
